# Improving Molecular Detection of Tick-Borne Pathogens in Citizen-Collected Ticks

**DOI:** 10.3390/pathogens15030310

**Published:** 2026-03-12

**Authors:** Andrea Matucci, Salvatore Scarso, Graziana Da Rold, Federica Obber, Filippo Marzoli, Andrea Ragusa, Fabio Formenti, Davide Treggiari, Antonio Mori, Cristina Mazzi, Andrea Tedesco, Pietro Sponga, Giulia Bertoli, Lucia Moro, Concetta Castilletti, Carlo Vittorio Citterio, Dora Buonfrate, Federico Giovanni Gobbi, Francesca Perandin, Chiara Piubelli

**Affiliations:** 1 Department of Infectious, Tropical Diseases and Microbiology, IRCCS Sacro Cuore Don Calabria Hospital, Negrar di Valpolicella, 37024 Verona, Italy; salvatore.scarso@sacrocuore.it (S.S.); andrea.ragusa@sacrocuore.it (A.R.); fabio.formenti@sacrocuore.it (F.F.); davide.treggiari@sacrocuore.it (D.T.); antonio.mori@sacrocuore.it (A.M.); andrea.tedesco@sacrocuore.it (A.T.); spongyptr94@gmail.com (P.S.); giulia.bertoli@sacrocuore.it (G.B.); lucia.moro@sacrocuore.it (L.M.); concetta.castilletti@sacrocuore.it (C.C.); dora.buonfrate@sacrocuore.it (D.B.); federico.gobbi@sacrocuore.it (F.G.G.); francesca.perandin@sacrocuore.it (F.P.); chiara.piubelli@sacrocuore.it (C.P.); 2 Istituto Zooprofilattico Sperimentale delle Venezie (IZSVe), Centro Specialistico Fauna Selvatica, SCT2-Belluno, 32100 Belluno, Italy; gdarold@izsvenezie.it (G.D.R.); fobber@izsvenezie.it (F.O.); fmarzoli@izsvenezie.it (F.M.); ccitterio@izsvenezie.it (C.V.C.); 3 Clinical Research Unit, IRCCS Sacro Cuore Don Calabria Hospital, Negrar di Valpolicella, 37024 Verona, Italy; cristina.mazzi@sacrocuore.it; 4 Department of Clinical and Experimental Sciences, University of Brescia, 25121 Brescia, Italy

**Keywords:** ticks, tick-borne pathogens, CE-IVD multiplex real-time PCR, zoonoses, citizen science

## Abstract

This study aimed primarily to evaluate the performance of two Conformité Européenne—In Vitro Diagnostic (CE-IVD) multiplex real-time PCR (rt-PCR) assays for the molecular identification of tick-borne pathogens (TBPs) of human interest on ticks removed from human skin and collected through a citizen science-based approach. As a secondary objective, the aggregated results were used to describe tick species distribution, developmental stages, and seasonal TBP circulation in 2024 in the considered area. The comparison was conducted on 116 tick samples collected in 2024 voluntarily delivered to a hospital in northeastern Italy. Detected TBPs were further confirmed with in-house-validated PCR methods and, where applicable, resolved to the species level. Clinically relevant pathogen species were identified as single infections or coinfections. Overall, 33.6% of tick samples tested positive for at least one TBP, and 6.9% showed coinfections. Kit B exhibited a higher detection rate for *Borrelia* spp. and *Rickettsia* spp. targets, partly reflecting its broader diagnostic specificity, while statistically significant differences in cycle threshold values were observed for *Anaplasma phagocytophilum* detection. The most frequently involved ticks were *Ixodes ricinus* nymphs, and the most represented area was Verona province. Late spring and early summer were identified as the periods with the highest tick conferment and pathogen diversity. Overall, the results support the use of multiplex real-time PCR commercial kits combined with citizen science-based tick collection as an effective approach for both diagnostic screening and regional surveillance of circulating ticks and TBPs.

## 1. Introduction

Ticks are among the most important blood-feeding ectoparasites affecting humans, wildlife, and domestic animals. Their presence and distribution are determined by environmental variables, host availability, and climatic factors [1]. Ticks are efficient vectors of multiple bacterial pathogens (e.g., *Borrelia* spp., *Rickettsia* spp., *Ehrlichia* spp., *Anaplasma* spp., *Coxiella burnetii*), intraerythrocytic protozoa (e.g., *Babesia* spp.), and neuroinvasive viruses from the Flavivirus genus (e.g., tick-borne encephalitis virus, TBEV). Those are transmissible from animal reservoirs to incidental human hosts, causing tick-borne diseases (TBDs) such as Lyme borreliosis (LB), spotted fever group (SFG) rickettsioses, anaplasmosis, ehrlichiosis, and tick-borne encephalitis (TBE) [2]. Currently, LB and TBE are the most prevalent TBDs in Europe [3,4]. TBEV exerts a significant public health impact in northern Italian regions such as Veneto, Friuli-Venezia Giulia, and Trentino-Alto Adige [5], where its distribution remains typically focal. Tick-borne pathogens (TBPs) such as bacteria may persist for a long time in ticks because they can be transmitted from stage to stage (trans-stadial transmission), from females to their offspring (vertical transmission), and from tick to tick via the host (horizontal transmission), depending on the pathogen [6].

TBPs pose significant medical, veterinary, and economic challenges worldwide, and are generally recognized as a critical “One Health” issue requiring an integrated approach [7]. Detection of TBPs in ticks removed from humans is increasingly utilized as a surveillance tool for monitoring the distribution of both vectors and associated pathogens [8]. This approach, supported by citizen science initiatives, enables cost-effective, reliable monitoring of TBP circulation and the direct estimation of exposure risk.

In tick-bitten symptomatic individuals, the direct molecular detection of the etiological agent is particularly informative during the acute phase of infection, since it can support clinical diagnosis in a phase in which serological tests often yield negative results. As summarized by Springer et al. [9], direct detection through molecular tests, such as nucleic acid amplification techniques (NAATs), offers greater sensitivity than traditional approaches, such as microscopy and pathogen culture. They also deliver results far more rapidly, enhancing overall diagnostic efficiency and enabling the detection of pathogens at low titers in diverse biological matrices, including ticks [9]. Multiplexed NAAT assays can also be employed as screening tools for the simultaneous detection and discrimination of a panel of TBP in a single sample assay [10]. In general, multiplexed assays offer superior turnaround times over single-target PCRs. This allows for more rapid detection and earlier intervention following exposure to tick-borne agents. Molecular testing of clinical samples from symptomatic individuals may therefore provide informative support for accurate diagnosis. Moreover, complementary molecular analysis on biting ticks, when available, can facilitate surveillance studies, providing insights into potential exposure to tick-borne agents. However, only a limited number of studies describe the use of commercial kits. Most molecular tests are single-target laboratory-developed PCR-based methods (in house), offering either broad genus-level specificity or narrow species-level specificity, which informs their use according to the epidemiological context of tick-borne pathogen circulation [10]. Therefore, the adoption of Conformité Européenne—In Vitro Diagnostic (CE-IVD)-marked multiplex real-time PCR assays may represent a valuable option for rapid, “ready-to-use” multi-target screening, such as in routine diagnostic laboratories for detecting infecting and coinfecting pathogens of human interest [11,12,13].

From the environmental perspective, human exposure to tick bites is reported to occur not only in rural and forested environments but also within urban and periurban settings [14]. In northern Italy, the likelihood of tick bites in anthropized areas has increased over years, accompanied by expanding TBD incidence and distribution, likely driven by vector expansion associated with climate change [15,16]. Among arthropods, ticks of the genus *Ixodes* are the principal vectors for a greater diversity of pathogens than any other arthropod globally [17]; however, other tick genera have been reported as circulating in Italy and the Mediterranean basin, such as *Dermacentor*, *Haemaphysalis*, *Hyalomma*, and *Rhipicephalus* [18]. In particular, *Hyalomma marginatum* can also transmit the Crimean–Congo hemorrhagic fever virus (CCHFV) [19].

Since 2016, the presence of TBP has been detected in ticks removed from bitten subjects who were referred to the IRCCS Sacro Cuore Don Calabria Hospital in northeastern Italy [5,20]. In this context, pathogen species detection has been previously performed outside the conferring hospital site, using in-house NAAT methods executed by veterinary laboratories of Istituto Zooprofilattico delle Venezie (IZSVe).

The primary objective of this study was the evaluation of two different CE-IVD real-time PCR assays for the molecular identification of a panel of TBP of human interest. The comparison was performed using a set of tick samples retrieved from individuals that voluntarily delivered ticks to our hospital in 2024. The results obtained from commercial kits were further confirmed and eventually resolved to the species level by the IZSVe specialized laboratory, using validated in-house molecular tests. As a secondary objective, we described the collection of individual ticks according to species and developmental stage, and the tick-borne pathogens detected were then analyzed in relation to the reported location of tick bites and their monthly distribution across the involved northeastern Italian provinces. The results support citizen-based surveillance in our area.

## 2. Materials and Methods

### 2.1. Tick Collection

From 8 April to 2 November 2024, we collected ticks that were delivered by bitten subjects to the Department of Infectious, Tropical Diseases and Microbiology (DITM) of the IRCCS Sacro Cuore Don Calabria Hospital in Negrar di Valpolicella, Verona (Italy). The submitted ticks were found to be attached to human skin after outdoor activities. They were removed either at home by the individuals themselves or detached by healthcare personnel at the emergency room of the IRCCS Sacro Cuore Don Calabria Hospital and conferred to the laboratory within 1 h. All samples were subsequently stored at −80 °C in Tropica Biobank (BBMRI-eric ID: IT_1605519998080235) until further processing. Information on the geographical area where the tick or ticks bite occurred was collected through a questionnaire.

### 2.2. Tick Identification and Nucleic Acid Extraction

Before molecular analysis, ticks were morphologically identified to the lowest possible taxonomic level (from family to species) by the parasitology laboratory of the IRCCS Sacro Cuore Don Calabria Hospital under a Nikon SMZ645 stereomicroscope (Nikon, Tokyo, Japan), using dichotomous keys [21,22], according to their conservation status and anatomical integrity. When multiple ticks were submitted by the same individual, specimens were morphologically identified and subsequently pooled, ensuring that a single composite tick sample per subject was included in downstream molecular analyses. Samples were stored submerged in 1× Blue Buffer^®^ (Microgembio, Southampton, UK) at −80 °C until the analysis; the ticks’ total nucleic acids were isolated using a thermo-enzymatic extraction protocol with the RNAGem^®^ Kit (Microgembio, Southampton, UK), according to the manufacturer’s instructions for complex tissue extraction without final DNase I treatment. Briefly, arthropod samples were mechanically disrupted with a pestle (two engorged elements were treated with citric acid anticoagulant at a 0.38% final concentration), and an RNAGEM reagent was added to the suspension and incubated at 75 °C for 1 h in a thermal block (Thermo Scientific, Milan, Italy), followed by an inactivation step at 95 °C for 5 min. The resulting extract was then stored at 4 °C for the subsequent PCRs.

### 2.3. Tick Pathogen Detection by Commercial CE-IVD Real-Time PCR Kit A and Kit B

Two commercial panels of CE-IVD rt-PCR tests were used for pathogen target detection. The first panel (Kit A) included three different rt-PCR reaction mixes (Mikrogen GmbH, Neuried, Germany): *alpha*Cube Rickettsia/Borrelia, *alpha*Cube Ehrlichia (detecting *Ehrlichia* spp./*Anaplasma phagocytophilum*), and *alpha*Cube TBEV. Molecular targets were not specified in the Kit A inserts. Positive samples displayed an exponential amplification curve, but no cut-off cycle threshold (Ct) value was indicated in the manual for positivity assignment, so we arbitrarily set the positivity cut-off at the final amplification cycle (Ct < 45). The second panel (Kit B) was the VIASURE Tick-Borne Disease Real-Time PCR Detection Kit (Certest Bioetc, Zaragoza, Spain) composed of three different rt-PCR reaction mixes targeting *Borrelia*/*Anaplasma*/*Coxiella* (BAC), *Rickettsia/Babesia/Ehrlichia* (ERB), and TBEV (T). The reactions were carried out according to the manufacturer’s instructions. Positive samples had a clear exponential amplification curve and a shared cut-off Ct < 40 (set by the manufacturer). Both panels were run on a CFX96 Real-Time PCR System (BioRad, Milan, Italy) located at DITM. Although Kit A included an exogenous extraction DNA or RNA control, and Kit B only an amplification control, to ensure the effectiveness of the nucleic acid extraction for both of the two diagnostic kits, a separate rt-PCR targeting the mitochondrial 16S rRNA or β-actin gene was tested as an extraction process control [23,24].

### 2.4. Tick-Borne Pathogen Molecular Confirmation and Species Determination

All TBP-positive samples detected by the two commercial kits described in paragraph 2.3 were also tested by the Specialistic Wildlife Center (SWC) at IZSVe, section of Belluno (Italy), for molecular confirmation and/or characterization at the species level, as previously described [20]. Briefly, samples positive for *Borrelia* spp. and *Anaplasma phagocytophilum* with commercial kits were further confirmed by in-house TaqMan real-time PCR assays [20], whereas TBEV samples were tested using a TaqMan real-time RT-PCR assay (rt-RT-PCR) targeting viral RNA [23]. The identification of *Borrelia* genospecies was performed using species-specific TaqMan real-time PCR assays designed to discriminate among *B. miyamotoi*, *B. afzelii*, *B. garinii*, and *B. burgdorferi sensu stricto* [25,26,27]. Samples positive for the presence of *Rickettsia* spp. and *Babesia* spp. were analyzed using specific endpoint PCRs followed by Sanger sequencing (PCR-Seq) [20]. The target pathogens, genes, and methods employed are summarized in Table 1. The rt-PCR reactions were carried out using the QuantiNova Probe PCR Kit (Qiagen, Milan, Italy) on a QuantStudio™ 5 Real-Time PCR System (Thermo Fisher Scientific, Milan, Italy). Endpoint PCR reactions were performed using AmpliTaq Gold^®^ DNA Polymerase on a Veriti 96-well Thermal Cycler (Applied Biosystems, Milan, Italy). PCR products were analyzed to confirm the expected amplicon size (indicated in Table 1) and subsequently purified using ExoSAP-IT™ PCR Product Clean-Up Reagent (Thermo Fisher Scientific), according to the manufacturer’s instructions. Sanger sequencing reactions were performed using the same primers employed in PCR and the BigDye Terminator v3.1 Cycle Sequencing Kit (Life Technologies, Hong Kong, China). Sequencing products were purified using the Optima DTR 96-well plate (Resnova, Roma, Italy) and analyzed in both directions using the SeqStudio Genetic Analyzer (Applied Biosystems, Milan, Italy). Sequences were aligned using MEGA version 6 and compared with those available in GenBank using the Basic Local Alignment Search Tool (BLAST+2.17.0 http://blast.ncbi.nlm.nih.gov/Blast.cgi, last accessed on 24 March 2025). Species identification was assigned when sequence identity was ≥99% (GenBank accession number ID: OR148326 for *Rickettsia slovaca*; EU883092 for *Rickettsia monacensis*; KJ663750 for *Rickettsia helvetica*; KU351826 for *Babesia capreoli*).

### 2.5. Statistical Analysis

Descriptive statistics included the number of ticks, means, standard deviations, range for continuous outcomes, and absolute and relative frequencies for categorical outcomes. Missing data was reported using absolute numbers and percentages. For molecular analysis output of CE- IVD kits, we used paired Wilcoxon signed-rank tests to compare the threshold cycle (Ct) values of samples that tested positive with at least one of the two diagnostic kits (Kit A and Kit B) for the detection of *Borrelia* spp., *Rickettsia* spp., and *Anaplasma phagocytophilum* molecular targets. The null hypothesis was that there was no difference in Ct values between the two kits (two-sided test, H_0_: µ_A_ − µ_B_ = 0). The null hypothesis (H_0_) tested for each comparison was that the median difference between Kit A and Kit B measurements equaled zero. The alternative hypothesis (H_a_) stated that the difference was not zero. After the Wilcoxon signed-rank test, we calculated the effect size (ES) as rank-biserial correlation. ES is useful for giving the magnitude of the differences: ES values between 0 and 0.1 represent “very small”, between 0.1 and 0.29 represent “small”, between 0.3 and 0.49 “medium”, and above 0.5 “large” [28]. We used the Jamovi 2.5.3 program and GraphPad Prism 10.4.2 for statistical analyses.

## 3. Results

### 3.1. Characteristics of Collected Ticks

A total of 141 individual ticks were collected at the DITM. Morphological identification of the collected ticks and determination of their developmental stage are represented in Table 2. One *Hyalomma marginatum* adult female was conferred by a tourist returning from Elba Island (central Italy), and the *Aponomma* sp. tick was from a traveler returning from Malawi.

### 3.2. Results of CE-IVD Kits for TBP Molecular Detection on Tick Samples

**Figure 1:**
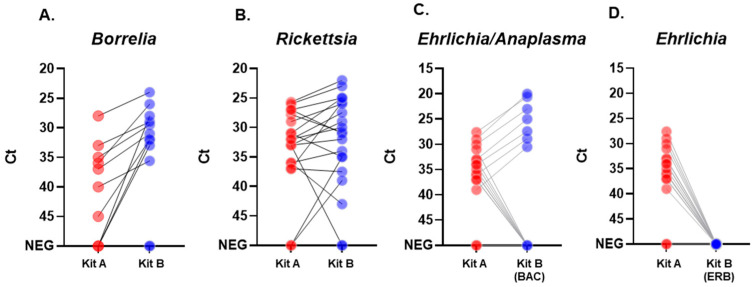
Slopegraph of the measured Ct obtained with the two kits on the same set of samples to detect *Borrelia* (**A**), *Rickettsia* (**B**), *Ehlichia* spp. vs. *Anaplasma phagocytophilum* (**C**), *Ehrlichia* spp. vs. *E. chaffeensis E. muris* (**D**) TBPs. Red dots indicate results obtained with Kit A, blue dots refer to Kit B, and a line connection indicates the same sample. The ERB mix of Kit B is specific for targeting *Ehrlichia chaffeensis* and *muris*, and the BAC mix is specific for detecting *Anaplasma phagocytophilum*.

Ticks were pooled by subject in 116 tick samples (tick distribution in tick samples is described in Supplementary Table S1). We compared the number of positive samples detected by the two kits, also evaluating the Ct output (Figure 1 and Table 3 and Supplementary Figure S1), given that the two kits may target different genes of the same pathogen. Table 4 summarizes the results obtained with Kit A and Kit B on the 116 tick samples analyzed. A cycle threshold value analysis is reported in Table 3, reporting the mean and range for the different kits and targets. Both kits shared positivity in 7 samples for *Borrelia* spp., 16 for *Rickettsia* spp., and 7 for *Ehrlichia* spp. with *Anaplasma phagocytophilum*. Considering the results from both kits, we detected a total of 10 positive samples for *Borrelia* spp., 19 for *Rickettsia* spp., 15 for *Ehrlichia* spp. (including 7 for *A. phagocytophilum*), and 2 for *Babesia microti/divergens*. For TBEV, in the unique positive sample, Kit A detected the virus with a high Ct value (Ct = 38), while Kit B registered a faint signal at the end of the cycling protocol (Ct = 45). None of the tested extracts were found to be positive for amplification of *Coxiella burnetii*, *Ehrlichia chaffeensis*, and *E. muris* molecular targets.

### 3.3. Confirmatory Testing and Molecular Characterization of the Detected TBPs

A third molecular method was used to confirm the presence of pathogen(s) in the tick samples that tested positive for TBPs using Kit A and/or Kit B and, when possible, to define the pathogen species by specific rt-PCR or conventional PCR followed by Sanger sequencing. Table 4 summarizes the results of species identification analysis for TBPs detected with Kit A and/or B. Only *Anaplasma phagocytophilum* was confirmed among the Anaplasmataceae-positive samples, due to the lack of an *Ehrlichia* spp. confirmatory test. We had a TBEV-positive PCR result from a sample using Kit A, but further identification was not successful due also to a low quantity of eluate. Four *Rickettsia* spp.- and one *Babesia* spp.-positive samples were confirmed but not typeable due to the low quality of the obtained sequences. Among spirochetes of the genus *Borrelia* (n = 10), the following species were detected: *B. afzelii* (n = 4), *B. burgdorferi* (n = 2), *B. garinii* (n = 2), and *B. miyamotoi* (n = 2); the latter was amplified only with Kit B, as indicated in the manufacturer’s datasheet specificity. Among the most frequently detected group of obligate intracellular bacteria of the genus *Rickettsia* (n = 19), we identified *R. helvetica* (n = 8), *R. monacensis* (n = 6), untypeable *Rickettsia* spp. (n = 4), and *R. slovaca* (n = 1). Within the *Ehrlichia* spp. group, *Anaplasma phagocytophilum* was confirmed in the seven samples positive for Kit B and Kit A, while eight additional samples positive only with Kit A were not detected as *Ehrlichia chaffeensis* and *E. muris* by Kit B and were not further characterized. The protozoan genus *Babesia* was detected in two samples, with one successfully typed as *B. capreoli*.

### 3.4. Statistical Comparison of CE-IVD Kits

We used paired Wilcoxon signed-rank tests to compare the Ct values of samples that tested positive for *Borrelia*, *Rickettsia*, and *Anaplasma phagocytophilum* molecular targets. The *Ehrlichia chaffeensis* and *muris* (mix ERB) and TBEV targets were not compared since no sufficient sample size of positive results was available for statistical analysis. For the *Borrelia* and *Rickettsia* targets, no statistically significant differences were observed between the Ct values obtained using Kit A and Kit B (*p* = 1.00 and *p* = 0.614 respectively), with very small and small effect sizes respectively (Table 5). Due to the broad specificity of Kit A for *Ehrlichia* spp., the statistical comparison with Kit B was restricted to samples confirmed as *Anaplasma phagocytophilum* by SWC testing. The difference between the two commercial kits was statistically significant for this target (Table 5, *p* < 0.05), with a very large effect size, indicating a substantial difference in Ct values between the two kits, with Kit B yielding lower Ct values (Supplementary Table S2).

### 3.5. TBPs Circulating as Coinfections

Overall, 47 TBPs were identified in 39 positive tick samples, eight of which were coinfected with two TBPs. As detailed in Table 6 and Supplementary Table S3, six different combinations of coinfecting TBP pairs were identified among all tick samples. Half of the coinfected samples consisted of two or more pooled tick elements; the pathogens most frequently involved were *Rickettsia* spp. (6/8, 75%) and *Ehrlichia* spp. (4/8, 50%). All *Babesia*-positive samples were detected in dual coinfections (2/2, 100%).

### 3.6. Provincial and Monthly Distribution of TBPs

**Figure 2:**
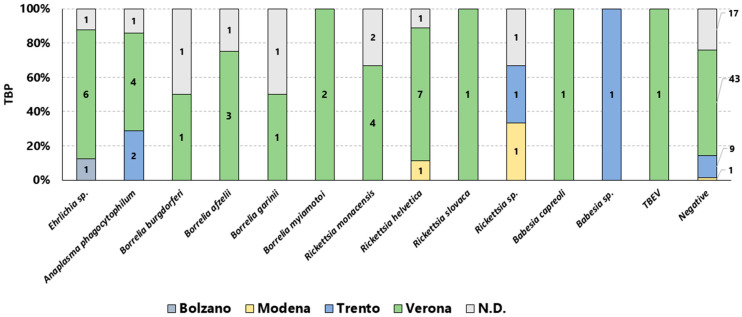
The 100% stacked bar chart illustrates the provincial distribution of detected TBPs. Each bar represents a specific pathogen, and the colors indicate the province: Bolzano, Modena, Trento, Verona, and not determined (N.D.). The numbers inside represent the numbers of TBPs.

**Figure 3:**
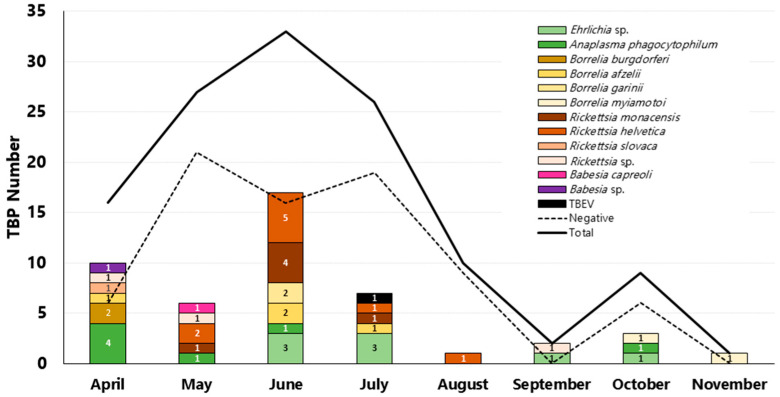
Monthly distribution of TBPs detected in 2024. The stacked bars represent the species and number of TBPs detected, the dashed line indicates TBP-negative samples, and the solid line refers to the total tested.

Tick bites were mostly from sites located in Verona province (58.6%, 68/116), followed by Trento–Bolzano provinces (11.2%, 13/116) and Modena (2.6%, 3/116). Information regarding the bite location was unavailable for 21.6% (25/116) of the submitted samples. We evaluated the collected data of detected TBPs according to the geographical locations where tick bites occurred and the seasonality. Figure 2 reports the different TBPs detected according to the provinces in which tick bites occurred; Verona province represents the wider pathogen presence with 31 TBPs detected, followed by Trento with 4, Modena with 2, and Bolzano with 1 detected. A graphical analysis illustrating the monthly trend of TBP detections for the year 2024 is presented in Figure 3.

## 4. Discussion

This study reports the detection performance evaluation of two different commercial multiplexed rt-PCR assays for the molecular detection of a panel of clinically relevant tick-borne pathogens (TBPs) within a hospital diagnostic setting. The evaluation was conducted using a set of 116 tick samples, with a predominance of the Ixodes genus, retrieved through citizen science-based surveillance of skin-removed ticks. A third, non-commercial, routinely used in-house test panel was employed at the veterinary laboratories of IZSVe to confirm positive results and to identify the specific species of TBPs. Further, the obtained results on TBPs were presented considering tick genera, developmental stage, province of origin, and the temporal distribution during the spring–autumn seasons.

Considering *Borrelia* and *Rickettsia* targets, Kit B showed a higher detection rate for both pathogens, but no statistically significant differences were recorded in the cycle threshold value. For *Borrelia*, the higher detection rate of Kit B is partially due to its broader diagnostic specificity toward also *Borrelia* of the relapsing fever group (BRFG) compared to Kit A, as indicated in the kit description and verified by our laboratory using external quality control sets (Quality Control for Molecular Diagnostics, QCMD, Glasgow, UK). BRFG species (*B. miyamotoi* and *hermsii*) have been associated with flu-like symptoms, recurrent fevers, and severe disease manifestations, including central nervous system infections and meningoencephalitis in immunocompromised patients [29]. *Borrelia miyamotoi*, transmitted by *I. ricinus*, has been documented to circulate in northern Italy [30,31,32], as we also confirmed its presence in our set of analyzed ticks, in particular in tick samples of Verona province. By contrast, we did not detect any *B. hermsii* or its soft-tick vector (*Ornithodoros hermsii*), but this is in line with the geographic distribution [33] in the New World. A total of 80% (8/10) of *Borrelia* detections in our analyzed samples were attributable to genospecies within the *Borrelia sensu lato* complex (*B. afzelii*, *B. burgdorferi*, and *B. garinii*), which are known to be pathogenic to humans. Our study unveiled the predominance of *B. afzelii* circulation in tick samples, also confirming previous findings [20].

The detection rate of *Rickettsia* was similar for the two kits (17 vs. 18); however, Kit A failed to detect two *Rickettsia* sp. samples returned by Kit B with high Ct values (Ct 33 and 39), while detecting one additional *R. helvetica* sample at Ct 33 that Kit B failed to identify. Among the overall 19 Rickettsia-positive samples, a relatively high proportion was identified as *R. helvetica* (9/19, 47%) and *R.* monacensis (6/19, 31.6%). Both species are emerging pathogens associated with spotted fever-like illness in humans, with documented cases also reported in Italy [34]. One *Rickettsia slovaca*, an emerging RFG pathogen, was detected. This bacterium is thought to circulate among small mammals and wild boars (*Sus scrofa*), the latter of which has been suggested as a reservoir in a recent study conducted in the Euganean Regional Park (Padova province, Veneto region) [35]. In humans, *R. slovaca* infection is associated with a specific clinical syndrome defined as “Scalp Eschar and Neck Lymphadenopathy after Tick bite” (SENLAT). In Italy, only six microbiologically confirmed cases of SENLAT were documented between 1996 and 2021, with *R. slovaca* and *R. massiliae* identified as the etiological agents through molecular analyses. In addition, ten further SENLAT cases were reported in Tuscany during the period of 2015–2022 [36].

Considering the *Anaplasma phagocytophilum* target, Kit B showed significantly better performance in terms of Ct output. Notably, *A. phagocytophilum* is of significant public health concern, as it is the causative agent of human granulocytic anaplasmosis (HGA), a disease characterized by infection of neutrophil cells [37,38]. We did not record *Ehrlichia chaffeensis* and *E. muris* positivity with Kit B. Only one case of human ehrlichiosis associated with mild symptoms caused by *E. canis* in a patient bitten by an engorged female *Haemaphysalis punctate* from south Italy was reported [39]. Notably, Kit A, as reported by the datasheet, can detect a broader range of *Ehrlichia* species of veterinary interest, such as *E. ewingii*, *E. canis*, or *Candidatus Neoehrlichia mikurensis* [18]. *Ehrlichia* spp. are primarily associated with canine tick-borne infections, which can occasionally cause severe complications in dogs.

Two tick samples tested positive for protozoa of the *Babesia microti*/*divergens* group by Kit B; in fact, DNA sequencing of 18S rRNA attributed one of these two samples to *Babesia capreoli*, which is a closely related species [40]. In Europe, where most infections are caused by either *B*. *divergens* or *B*. *venatorum*, only about fifty human cases have been reported since 1957 [41]. The Rickettsia-like bacterium *Coxiella burnetii*, the etiologic agent of Q-fever, was not detected in any of the ticks analyzed.

Tick-borne encephalitis virus was presumably identified in one tick sample of Verona province, reinforcing recent findings of TBEV circulation in this area [20]; however, the results remain inconclusive because no confirmation was obtained.

The overall results indicated that Kit B seems to perform better as a screening tool for shared TBPs of human interest, with the exception of TBEV, for which no conclusions can be drawn. However, Kit B could also detect a broader range of pathogen species in the same number of rt-PCR mixes compared to Kit A in the tested configuration.

Among the retrieved ticks, 66.4% (77/116) tested negative for all TBP targets with both kits; the remaining 33.6% (39/116) were found to be infected by a single TBP (N = 31, 26.7%) or by two TBPs (N = 8, 6.9%). The overall percentage of infected ticks found in this study more than doubled compared to that reported recently by Moro L. et al. [20] in the same area from 2019 to 2022. This may indicate a substantial rise in pathogen circulation, although the present study could also reflect better performance in the detection protocol and different conferment frequencies. *Babesia*, *Rickettsia*, and *Ehrlichia* sp. were the most frequently coinfecting pathogens detected. All detections of *Babesia* occurred as part of coinfections, underscoring its consistent association with other pathogens (*Anaplasma phagocytophilum* or *Rickettsia helvetica*).

The results obtained from this molecular analysis were coupled with the morphological examination of the tick collection in order to monitor tick species, developmental stages, and the circulation of tick-borne pathogens (TBPs) within the geographic area covered by the collected ticks, as well as throughout the annual period during which they were detected in the entire warmer season of 2024.

A total of 19.1% (27/141) of ticks had key structures either missing or deformed, making it impossible to accurately match them with established dichotomous keys, and they were described generally as hard ticks of Family Ixodidae subfam. Ixodinae. The collection revealed a predominance of *Ixodes ricinus* (51.7%, 73/141), confirming its extensive distribution across the northern Italy geographical area. The overall most represented developmental stage was the nymphal stage (70.9%, 100/141), followed by the female adult stage (21.3%, 30/141). Feeding status was generally not recorded; only two ticks were reported as fully engorged. The predominance of nymph life stage aligns with the life cycle dynamics, in terms of the abundance and activity of *I. ricinus* [42]. This is epidemiologically relevant, as many infections are primarily transmitted during periods of larval and nymphal activity between late spring and late summer [43]. Considering coinfected samples, most of them were at the nymphal stage. Tick-borne coinfections arise when ticks acquire multiple pathogens during feeding on coinfected hosts or sequential blood meals during different life stages, potentially exacerbating disease severity and leading to misdiagnosis or inappropriate treatment [44]. The *Dermacentor marginatus* tick was collected in a periurban hilly area of Verona and tested positive for *R. slovaca*. This tick species was recently documented in northern Italian regions [45] and recognized as a vector of this SFG *Rickettsia* [46]. With regard to *Hyalomma marginatum*, the presence of one of the main vectors of Congo–Crimea hemorrhagic fever (CCHF) confirms that Italy, along with other European countries, has been considered to be at medium risk for the circulation of CCHF, based on a recent risk assessment [47]. This sample was further tested for the presence of the CCHF viral genome, yielding a negative result.

Tick bite sites were mainly located in northeastern Italian regions, encompassing different ecological and geographic settings, mostly within the province of Verona (58%) and extending to the northern provinces of Trento and Bolzano (11.2%); however, samples were also collected from other Italian provinces (i.e., Modena, Forlì-Cesena, Trieste, Livorno) and extra-Italian sites. Considering all TBPs detected, 66% (31/47) were located in Verona province and 14.9% (7/47) in Trento, Bolzano, and Modena provinces. The remaining 19.1% (9/47) were located in other undisclosed geographic locations.

From a monthly perspective, submission data showed that citizen participation in tick sample delivery was highest between May and July. During this period, TBP detections accounted for 64% of the total pathogens identified in all collected samples (30/47). The highest proportion of *Anaplasma phagocytophilum* detections was observed in April (57%, 4/7). Nearly half of all *Rickettsia* spp. detections (47%, 9/19) occurred in June. *Borrelia miyamotoi* was detected exclusively in October and November (100%, 2/2). Tick populations can also be influenced by climatic events [48], and according to Italian meteorological surveillance data [49], rainfall in the Veneto region during May, September, and October 2024 doubled the 30-year seasonal precipitation average, influencing outdoor activities and tick bite exposure. The detected prevalence of TBPs was consistent with findings from alternative surveillance methodologies in the same geographical region, supporting the reliability and effectiveness of our operational protocol for monitoring tick-borne pathogens [50].

Compared to active entomological surveillance methods (e.g., dragging and flagging of questing host-seeking ticks), collecting ticks from bitten citizens provides a more accurate assessment of exposure risks across multiple environments, whereas active surveillance assesses actual tick density.

This study presents several limitations that should be considered when interpreting the results. First, although both kits were composed of three different RT-PCR mixes and shared targets for the principal human-pathogenic TBPs (*Borrelia*, *Rickettsia*, *Ehrlichia/Anaplasma*, and TBEV), their target panels were not completely overlapping, and the gene targets were not known in some cases. Kit B additionally detected *Babesia* spp. and *Coxiella burnetii*, whereas these targets were not included in the tested configuration of Kit A. Although equivalent reagents are currently available for Kit A, they require additional RT-PCR mixes and were not evaluated in this study. Consequently, direct comparability between assays is only partial. In addition, the small sample size further limits statistical power, and no definitive conclusions regarding differences in positivity rates can be drawn.

Second, tick collection relied on voluntary citizen submission. This surveillance approach may have resulted in the uneven geographic and temporal distribution of samples. Submission rates likely reflect public awareness and seasonal outdoor activity rather than true tick density or pathogen prevalence. Furthermore, reliance on patients to recall the probable location of tick bites introduces potential geographic misclassification. The implementation of a structured and continuous information campaign targeting general practitioners or hiking clubs could improve awareness in the population, resulting in a more conscious participation. Moreover, promoting the use of passive data collection methods, such as a smartphone app, could facilitate people’s engagement and provide a more precise geolocation of ticks.

Third, in some cases, the species or developmental stage of ticks was not identifiable, limiting the ecological interpretation of pathogen distribution and vector competence.

Fourth, the absence of clinical data from individuals bitten by infected ticks restricts assessment of the clinical relevance of detected TBPs. Further studies focusing on the real transmission rate of pathogens from ticks to the bitten subjects could assess the correlation between TBPs detected in ticks and subsequent human diseases.

Finally, in cases where multiple ticks were removed from the same individual, pooled molecular analysis was performed. While practical, this approach may underestimate individual tick infection rates and obscure coinfection dynamics. Overall, larger prospective studies integrating standardized molecular panels, expanded pathogen coverage, clinical follow-up, and systematic geographic documentation would strengthen epidemiological interpretation and improve risk assessment.

The application of a citizen science-based surveillance approach may enhance TBD prevention policies by enabling the early detection of pathogen circulation, improving geographic risk mapping, promoting public awareness, and supporting cost-effective, area-specific interventions within a One Health framework, enhancing diagnostic workflows. Studies involving bitten individuals should evaluate the clinical significance of these findings, especially in cases with low pathogen loads.

## 5. Conclusions

This study demonstrates that multiplex CE-IVD rt-PCR assays are effective tools for detecting TBP in real-world surveillance, with Kit B offering superior screening performance thanks to its broader species coverage within the same reaction mixes. The analysis confirms the active circulation of several clinically relevant zoonotic agents, particularly *Borrelia*, *Rickettsia*, and *Anaplasma phagocytophilum*, with a notable prevalence of *Ixodes ricinus* nymphs and frequent coinfections during late spring and early summer. The integration of citizen science based tick collection with molecular diagnostics proved to be a reliable approach for assessing human exposure risk and monitoring seasonal and geographic trends in tick-borne pathogen circulation.

## Figures and Tables

**Table 1:** Summary of the methods used for molecular analysis at IRCCS Sacro Cuore Don Calabria Hospital (ISCDC) or IZS delle Venezie (IZSVe) laboratories.

Genus	Test/Kit	Method	Laboratory	Gene Target	Pathogen Target
*Borrelia*	Mikrogen *alpha*Cube Borrelia/Rickettsia (Kit A)	rt-PCR	ISCDC	Not available	*Borrelia burgdorferi s.lato*
	Certest Mix BAC (Kit B)	rt-PCR	ISCDC	*23S rRNA*	*Borrelia* spp. (including *B. miyamotoi* and *B. hermsii*)
	In house (SWC)	rt-PCR	IZSVe	*23S rRNA*	*Borrelia* spp.
				*p41*	*B. miyamotoi*
				*flaB*	*B. afzelii*, *B. garinii*, *B. burgdorferi s.stricto*
*Rickettsia*	Mikrogen *alpha*Cube Borrelia/Rickettsia (Kit A)	rt-PCR	ISCDC	Not available	*Rickettsia* spp.
	Certest Mix ERB (Kit B)	rt-PCR	ISCDC	*23S rRNA*	*Rickettsia* spp.
	In house (SWC)	PCR-Seq (511 bp)	IZSVe	*rOmp*	*Rickettsia* spp.
*Ehrlichia*	Mikrogen *alpha*Cube Ehrlichia (Kit A)	rt-PCR	ISCDC	Not available	*Ehrlichia* spp., *Anaplasma* spp.
	Certest Mix ERB (Kit B)	rt-PCR	ISCDC	*GroEL*	*E. chaffeensis*, *E. muris*
	Certest Mix BAC (Kit B)	rt-PCR	ISCDC	*msp2*	*A. phagocytophilum*
	In house (SWC)	rt-PCR	IZSVe	*msp2*	*A. phagocytophilum*
*Babesia*	Certest Mix ERB (Kit B)	rt-PCR	ISCDC	*CCT-eta*/*hsp70*	*Babesia microti*, *Babesia divegens*
	In house (SWC)	PCR-Seq (500 bp)	IZSVe	*18S rRNA*	*Babesia* spp.
*Coxiella*	Certest Mix BAC	rt-PCR	ISCDC	*IS1111*	*Coxiella burnetii*
TBEV	Mikrogen *alpha*Cube TBE (Kit A)	rt-RT-PCR	ISCDC	Not available	TBEV
	Certest Mix T (Kit B)	rt-RT-PCR	ISCDC	*3′-UTR*	TBEV
	In house (SWC)	rt-RT-PCR	IZSVe	*3′-UTR*	TBEV

Abbreviations: rt-PCR, real-time PCR; rt-RT-PCR, one-step real-time reverse transcription PCR; PCR-Seq, endpoint PCR followed by amplicon sequencing. Expected amplicon sizes are indicated in base pairs (bp); BAC: Kit B mix targeting *Borrelia*/*Anaplasma*/*Coxiella*; ERB: Kit B mix targeting *Rickettsia*/*Babesia*/*Ehrlichia;* T: Kit B mix targeting TBEV; SWC, Specialistic Wildlife Center at IZSVe. *18S rRNA*: 18S ribosomial RNA; *p41* and *flaB*: flagellin genes; *rOmpB*: Rickettsial Outer-Membrane Protein B gene; *UTR*: untranslated region; *msp2*: major surface protein 2 gene; *GroEL*: chaperonine GroEL gene; *CCT-eta*: Chaperonin Containing T-Complex Polypeptide Subunit Eta gene; *hsp70*: heat shock protein 70 gene; *IS1111*: insertion sequence.

**Table 2:** Developmental stage distribution and relative abundance of ticks collected and identified during the study.

Tick Species	Larvae		Nymph		Adult (F)		Adult (ND)		Total	
	N	%	N	%	N	%	N	%	N	%
Family Ixodidae subfam. Ixodinae	3	2.1	19	13.5	1	0.7	4	2.8	27	19.1
*Ixodes* spp.	3	2.1	22	15.6	2	1.4	-	-	27	19.1
*Ixodes ricinus*	1	0.7	52	36.9	20	14.2	-	-	73	51.7
*Ixodes acuminatus*	-	-	6	4.3	5	3.5	-	-	11	7.8
*Dermacentor marginatus*	-	-	-	-	1	0.7	-	-	1	0.7
*Aponomma* sp.	-	-	1	0.7	-	-	-	-	1	0.7
*Hyalomma marginatum*	-	-	-	-	1	0.7	-	-	1	0.7
Total	7	5.0	100	70.9	30	21.3	4	2.8	141	100

Abbreviations: ND, unidentified adult; F, adult female; N, number of identified elements; %, percentages of the identified elements calculated on the total number of ticks morphologically analyzed (N = 141).

**Table 3:** Molecular detection performances by the two CE-IVD kits based on the number of amplified samples and Ct values of TBP identified. The percentage of positive ticks for each pathogen genus and the corresponding kit used refer to the 116 tested samples.

Target/Assay	Positive Samples (N, %)	Ct Mean ± Stdv	Ct Range (Min–Max)
*Borrelia* Kit A	7 (6%)	36.3 ± 5.3	17 (28–45)
*Borrelia* Kit B	10 (8.6%)	30.0 ± 3.4	11.6 (24–35.6)
*Borrelia* Overall	10 (8.6%)	-	-
*Rickettsia* Kit A	17 (14.7%)	31.4 ± 3.8	11.3 (25.7–37)
*Rickettsia* Kit B	18 (15.5%)	30.6 ± 5.8	21 (22–43)
*Rickettsia* Overall	19 (16.4%)	-	-
*Ehrlichia* spp. Kit A	15 (12.9%)	33.8 ± 3.3	11.4 (27.6–39)
*Anaplasma phagocytophilum* Kit B	7 (6%)	25.1 ± 4.1	10.5 (20–30.5)
TBEV Kit A	1 (0.9%)	38	-
TBEV Kit B	0 * (0.9%)	45	-
TBEV Overall	1 * (0.9%)	-	-
*Babesia microti/divergens* Kit B	2 (1.7%)	33.5 ± 7.8	11 (28–39)

* Equivocal: this sample presented a faint amplification curve.

**Table 4:** Comparison of TBP species detected in tick samples by the two commercial kits, as confirmed by the third SWC test. Discordant results were indicated with the Ct recorded.

	Overall Total	Kit A	Kit B	Discordant Samples (Ct Kit A vs. Ct Kit B)
*Borrelia myiamotoi*	2	0	2	2 (NA vs. 28; NA vs. 33)
*Borrelia garinii*	2	2	2	0
*Borrelia burgdorferi*	2	1	2	1 (ND vs. 32)
*Borrelia afzelii*	4	4	4	0
*Rickettsia* sp. °	4	2	4	2 (ND vs. 35; ND vs. 39)
*Rickettsia slovaca*	1	1	1	0
*Rickettsia monacensis*	6	6	6	0
*Rickettsia helvetica*	8	8	7	1 (33 vs. ND)
*Anaplasma phagocytophilum*	7	7	7	0
*Babesia* sp. °	1	0	1	1 (NA vs. 39)
*Babesia capreoli*	1	0	1	1 (NA vs. 28)

Abbreviations: NA: target not available; ND: not detected; °: amplified band of *Rickettsia* sp. and *Babesia* sp. resulted in no readable sequence.

**Table 5:** Wilcoxon paired samples test results comparing Ct values between two kits (Kit A vs. Kit B) for detection of TBP. The table reports *p*-values and effect sizes (ESs) for three pathogen groups: *Borrelia*, *Rickettsia*, and *A. phagocytophilum*.

Kit A vs. Kit B		*p*	ES
*Borrelia* [Kit A]	*Borrelia* [Kit B]	1.00000	0.0182 (very small)
*Rickettsia* [Kit A]	*Rickettsia* [Kit B]	0.61451	0.137 (small)
*A. phagocytophilum* * [Kit A]	*A. phagocytophilum* [Kit B]	0.02225	1.00 (large)

*: Analyses were performed using values of *A. phagocytophilum* samples (confirmed by SWC testing).

**Table 6:** Composition of TBP-coinfected tick samples by developmental stage and species.

Pathogen 1	Pathogen 2	Tick Sample Composition
*Rickettsia* sp.	*Borrelia burgdorferi* sp.	1 Adult (F) of *I. ricinus*
*R. helvetica*	*Babesia capreoli*	1 Adult (F) of *I. ricinus*
*R. monacensis*	*Ehrlichia* sp.	Pool of five nymphs of Family Ixodidae subfam. Ixodinae
*R. monacensis*	*Ehrlichia* sp.	One nymph of Family Ixodidae subfam. Ixodinae
*R. monacensis*	*Ehrlichia* sp.	One nymph of *Ixodes* spp.
*R. helvetica*	*A. phagoc* *ytophilum*	Pool of five nymphs of Family Ixodidae subfam. Ixodinae
*B. afzelii*	*Ehrlichia* sp.	Pool of two nymphs of Family Ixodidae subfam. Ixodinae
*A. phagocytophilum*	*Babesia* sp.	Pool of three nymphs/three adults (F) of *I. acuminatus*

## Data Availability

The dataset for this study is available upon request in Zenodo at the following link: http://doi.org/10.5281/zenodo.18411176, accessed on 3 March 2026.

## Supplementary Materials

The following supporting information can be downloaded at https://www.mdpi.com/article/10.3390/pathogens15030310/s1. Supplementary Figure S1. Scatter plot graph that compares the Ct results of the two real-time PCR kits across different TBP targets. Red dots indicate results obtained with Kit A and blue dots refer to results obtained with Kit B. The bar represents the mean and standard deviation of the Ct values; Supplementary Table S1. Composition of tick samples analyzed from 8 April 2024 to 2 November 2024; Supplementary Table S2. CE-IVD kit performance limited to the *Anaplasma phagocytophilum* target indicated by SWC test; Supplementary Table S3. Frequency analysis of TBP mono and coinfected ticks.

## Abbreviations

The following abbreviations are used in this manuscript:

CE-IVD	Conformité Européenne In Vitro Diagnostic
DEET	Diethyltoluamide
TBEV	Tick-borne encephalitis virus
TBDs	Tick-borne diseases
LB	Lyme borreliosis
SFG	Spotted fever group
VBD	Vector-borne disease
