# Supplementary material for: Improving Molecular Detection of Tick-Borne Pathogens in Citizen-Collected Ticks
Source: Pathogens. 2026 Mar 12;15(3):310. doi: 10.3390/pathogens15030310 (PMC13029150; doi:10.3390/pathogens15030310)
Supplement: Supplementary file 1 [file pathogens-15-00310-s001.zip › pathogens-4160109-supplementary.pdf]

	N
**Total ticks collected**	141
**Single tick samples**	104
**Pooled ticks samples**	37
**Total tick samples analyzed**	116

Supplementary Table S1. Composition of tick samples analyzed from 8 April 2024 to 2 November 2024.

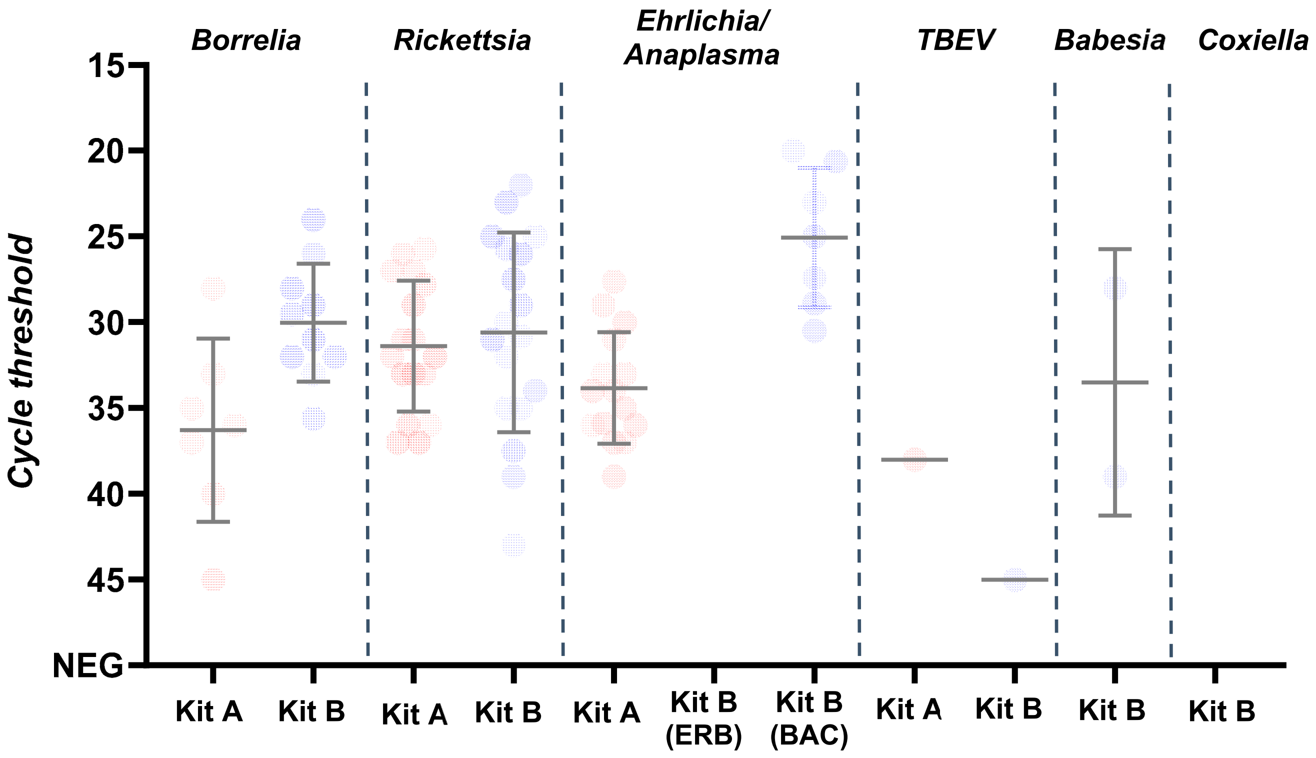

Supplementary Figure 1. Scatter plot graph that compares the Ct results of the two real-time PCR kits across different TBP targets. Red dots indicate results obtained with Kit A and blue dot refers to Kit B. Bar represent mean and standard deviation of the Ct values.

Target / Assay	Positive samples (N, %)	Ct Mean ± Stdv	Ct Range (min–max)
SWC confirmed *Anaplasma phagocytophilum* Kit A –*Ehrlichia* spp.	7 (6%)	32.7±4.1	11.4 (27.6-39)
SWC confirmed *Anaplasma phagocytophilum* Kit B - mix BAC	7 (6%)	25.1±4.1	10.5 (20-30.5)

Supplementary Table S2. CE-IVD kit performance limited to the *Anaplasma phagocytophilum* target indicated by SWC test. The percentage of positive ticks for each pathogen genus and the corresponding kit used are referred to 116 tested samples. Abbreviation: SWC, Specialistic Wildlife Center at IZSVe

Sample ratio	Fraction	Percentage
Single infected tick samples/TBP positive tick samples	31/39	79.5 %
Dual infected tick samples/TBP positive tick samples	8/39	20.5 %
Dual infected tick samples/Tick samples ≥ 2 elements	4/8	50 %
Dual infected tick samples containing *Rickettsia* (all) /All Dual infected tick samples	6/8	75 %
Dual infected tick samples containing *Ehrlichia* spp. /All Dual infected tick samples	4/8	50 %
Dual infected tick samples containing *Babesia* (all) / All *Babesia* infected tick samples	2/2	100 %

Supplementary Table S3. Frequency analysis of TBP mono and coinfected ticks.
